# micRocounter: Microsatellite Characterization in Genome Assemblies

**DOI:** 10.1534/g3.119.400335

**Published:** 2019-08-02

**Authors:** Johnathan Lo, Michelle M. Jonika, Heath Blackmon

**Affiliations:** Department of Biology; Texas A&M University; College Station, TX 77843

**Keywords:** microsatellite, genome analysis, genomics, repetitive sequences

## Abstract

Microsatellites are repetitive DNA sequences usually found in non-coding regions of the genome. Their quantification and analysis have applications in fields from population genetics to evolutionary biology. As genome assemblies become commonplace, the need for software that can facilitate analyses has never been greater. In particular, R packages that can analyze genomic data are particularly important since this is one of the most popular software environments for biologists. We created an R package, micRocounter, to quantify microsatellites. We have optimized our package for speed, accessibility, and portability, making the automated analysis of large genomic data sets feasible. Computationally intensive algorithms were built in C++ to increase speed. Tests using benchmark datasets show a 200-fold improvement in speed over existing software. A moderately sized genome of 500 Mb can be processed in under 50 sec. Results are output as an object in R increasing accessibility and flexibility for practitioners.

Genomes are composed of sequences that can be classified by their function, composition, or location on the chromosome. Microsatellites are DNA sequences that are characterized by the repetition of motifs between 2 and 6 bp. These sequences are primarily found in non-coding regions of the genome, although some are located in regulatory or intronic regions (Pearson *et al.* 2005). Microsatellites in non-coding regions are thought to be mostly free from selective pressure, and their evolution is therefore largely a stochastic function of time. One notable exception to this is microsatellites in regions upstream from genes or in introns where they can have impacts on modulating expression levels (Rohilla and Gagnon 2017).

Combined, these characteristics of microsatellites make them useful in a variety of applications. Their repetitive nature makes them easy to detect and characterize in genome sequence data. Their relative neutrality in comparison to many sequence classes allows them to serve as biological clocks on evolutionary timescales and allows for the inference of population demography (Nielsen 2005; Slatkin 1995; Spencer *et al.* 2000; Sun *et al.* 2009; Waits *et al.* 2000). In studies of natural populations, microsatellites are frequently used to assess genetic diversity within species and populations (Fischer *et al.* 2017; Serrano *et al.* 2009). The variability and abundance of microsatellites also allow them to be used to differentiate between individuals within a population, hence leading to applications in forensics, kinship analysis, and medical profiling (Detwiler *et al.* 2017; Schumer *et al.* 2017). Finally, microsatellite analysis has been used to monitor the progression of cancer through quantifying the rate at which microsatellites are gained, or lost (Boland *et al.* 1998; Sideris and Papagrigoriadis 2014; van Tilborg *et al.* 2012).

More than a tool, microsatellites themselves are also under investigation. Microsatellites typically have higher mutation rates than point mutations. The most widely accepted hypothesis for this high mutation rate is replication slippage, in which DNA polymerase may slip on the template strand leading to either an expansion or contraction of the number of repeat units present (Klintschar *et al.* 2004). The distribution of microsatellite lengths is thought to be maintained through a balance of point mutations which disrupt repeats and replication slippage (Kruglyak *et al.* 1998). Microsatellites upstream of genes or in intronic regions can impact gene regulation with phenotypic effects involving diseases like acute lymphoblastic leukemia or changes in diastolic blood pressure and albumin levels (Akagi *et al.* 2009; Gymrek *et al.* 2016). Large comparative studies characterizing microsatellite content of genomes across large clades have shown that some lineages show exceptionally rapid changes in both microsatellite content and abundance (Adams *et al.* 2016; Fan and Guo 2018). Microsatellite analyses have diverse applications and are themselves an important aspect of genome evolution. Thus, it is crucial to have efficient, accurate, and freely available tools for microsatellite characterization. Currently the most widely used program to our knowledge is Palfinder which is written in Perl and as such can be challenging for some users to install and run (Adams *et al.* 2016; Castoe *et al.* 2010; Castoe *et al.* 2012). Our R package, micRocounter, aims to resolve this challenge and facilitate accurate and fast microsatellite characterization. This software provides a range of information useful in different types of analyses, including total content of each type of microsatellite and their location in the genome. Users can install this open source R package directly from GitHub, and it is compatible with all major operating systems.

## Methods

We implemented our microsatellite characterization tool in R to provide the greatest utility to the greatest number of biologists. Compared to competing platforms, R provides a significant advantage in statistical computing and user interface. However, the convenience and flexibility of R is undermined by a lack of speed. Therefore, the bulk of the algorithm was written in C/C++ and ported to R via the package Rcpp (Eddelbuettel and Balamuta 2018). The C/C++ algorithm uses only functions included in ANSI C. The package has no other dependencies, and is only 94 KB.

The overall function of the package is to process FASTA files and output microsatellite information as R objects. The primary function is *ReadFasta*, which takes three arguments: *file*, *minrepeats*, and *squishy*. The argument *file* is supplied as a character vector and describes the relative location of the FASTA file to be analyzed. The argument *minrepeats*, is supplied as a numeric vector of length five, with integer values corresponding to the minimum number of twomers, threemers, fourmers, fivemers, and sixmers required to be counted as a microsatellite for the purposes of analysis. The argument *squishy* is also a numeric vector of length five and with integer values corresponding to the maximum number of imperfections in a microsatellite that can be encountered before the microsatellite is considered to have ended. The R object returned by *ReadFasta* is a list with seven elements. The first five elements are each lists that contain the microsatellite content information for each monomer length. These first five elements each contain four vectors that hold the sequence name, location, monomer type and number of repeats present in the analyzed FASTA file. The final two elements contain the assembly size and the total microsatellite content in Mb. A typical script showing usage is shown below:

 library(micRocounter)
 micro.analysis <- ReadFasta(file = “Chrysina_woodi”,
 minrepeats = c(6, 4, 3, 3, 3),
 squishy = c(1, 1, 1, 1, 1)
 twomer.report <- FindXmer(mon_len = 2,
 x = micro.analysis)

When ReadFasta is called, an internal C function reads the FASTA file character by character, omitting newlines and without regard to capitalization. Each character is read into a buffer string, consisting of the previous 12 characters. If a sequence of recent characters match the sequence immediately preceding it, a flag is triggered, the repeat sequence is temporarily stored, and subsequent sequences are compared to this stored sequence only. Once the number of repeated sequences exceeds the minimum number specified by the second argument (*minrepeats*), the location of the initial repeat is permanently stored, along with the sequence itself. Subsequent bases will continually be compared with the memorized sequence until the number of discontinuities exceeds the value specified by the third argument (*squishy*). At this point, the flag will reset, and the number of repeats included in the run is stored. The algorithm then begins anew and subsequent bases will be cycled through the buffer string until the flag is triggered again. The presence of ambiguous base calls in a sequence are dealt with by treating these bases as mismatches with regard to any repeat they are found within. For instance, a sequence of ATNTATATATAT would be recognized as an AT repeat of length 6 with one mismatch. Other ambiguous base calls such as R and Y are treated identically. This algorithm continues until the end of file is reached, at which point the location, length, and sequence descriptions for all repeats are assembled into the R list described above, along with total microsatellite content and genome size, and exported back to the original R function. Below we show pseudocode that describes the basic functioning of our algorithm.

### Pseudocode

 Open FASTA file as a file stream;
 while filestream != end of file{
 if filestream is a member of (A, G, T, C):{
 add filestream to a 12 character buffer;
 check buffer for repeats;
 if repeats are found for a monomer of length x:{
 flag on;
 continue reading file;
 if next sequence of x characters contains repeats:
 increment a counter and continue;
 else:
 store length and location in memory;
 flag off and get out;
 if number of mismatches exceeds threshold:
 store length and location in memory;
 flag off and get out;
 }
 }
 }

 Once a FASTA file has been processed, control reverts back to the R platform and additional analyses can be run. The package comes with one additional function, *FindXmers*, which takes the list object created by *ReadFasta* and reorganizes for simplicity and accessibility. It takes two arguments, *mon_len* and *micro_list*. The argument *mon_len* is a single integer from 2-6 and describes the subset of microsats that should be the organized into a table. The argument *micro_list* is the list object returned by the function *ReadFasta*. The function *mon_len* returns a dataframe with six columns. The row names denote the monomer. The first two columns provide the total number of loci and the total number of bases found in a given monomer class in the FASTA file. The third column contains a list giving the location, length and sequence for each microsatellite locus. The final three columns report the fraction that a monomer represents within a repeat length class, the fraction that a monomer represents for all microsatellites, and the fraction that a monomer represents in the whole genome.

For comparison we tested our software against Palfinder version 0.02.04. All analyses described below were conducted with FindPrimers set to 0, platform as 454, inputFormat as FASTA, and minimum repeats as 6,4,3,3,3 for two, three, four, five, and six-mers respectively. All other parameters were left as default, and input and output paths were set to local directories.

### Data Availability

The package micRocounter can be downloaded and installed direct from GitHub: github.com/johnathanlo/micRocounter, and scripts for running all analyses described in the paper are available from the GitHub repository github.com/johnathanlo/micRocounter_manuscript.

## Results and Discussion

**Figure 1:**
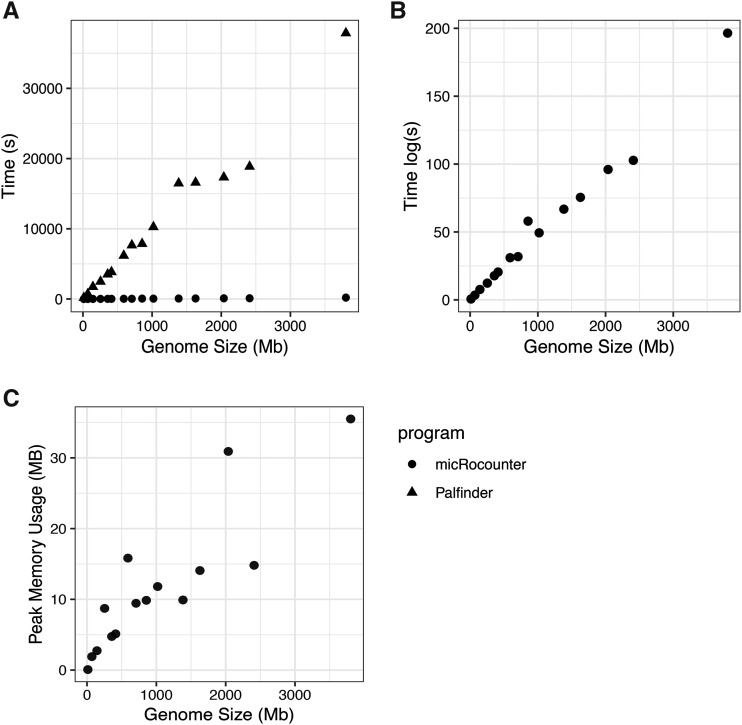
Processing time and memory usage of micRocounter across 15 representative genomes. In each panel the x axis represents genome size of the benchmark genomes in Mb. A) Comparison of execution time for micRocounter and Palfinder on benchmark genome set. B) Execution time for micRocounter on benchmark genome sets with time on a log scale. C) Peak memory usage running micRocounter on benchmark genomes.

To evaluate the accuracy of our software we characterized microsatellite content of a FASTA file generated with known microsatellite content. The simulated sequence was 28 Kbp long with no microsatellite loci. Within this sequence, we randomly inserted 117 microsatellite loci comprising a total length of 3626 bp. These loci were comprised of 23 two-mer loci (366 bp total), 22 three-mer loci (528 bp total), 24 four-mer loci (764 bp total), 24 five-mer loci (960 bp total), and 24 six-mer loci (1008 bp total). In a comparison of micRocounter and Palfinder (Castoe *et al.* 2012) microsatellite content was identified equally. The only difference in the result of the two programs was in the collapsing of equivalent repeats.(reversals and complimentary sequences). For instance, a repeat motif of GACT can be reported as TCAG, AGTC, or CTGA. To assess the efficiency of our software, we analyzed 15 insect genome assemblies and compared micRocounter run-times to Palfinder. The sizes of our 15 benchmark genomes ranged from 12 Mbp to 3.8 Gbp (Table 1). We found that our software offered considerable improvements in speed compared to Palfinder. Moreover, these improvements are substantial enough to render the actual processing time of genomes inconsequential in the majority of circumstances. All 15 genomes were analyzed by micRocounter in approximately 13 min (769 sec), compared to 42 hr (151572 sec) in Palfinder (Figure 1A-B). This equates to approximately a 200-fold improvement in runtime allowing for the analysis of large sets of genomes to be analyzed. Average genome processing speed for micRocounter was 20 Mbp/s, fast enough to analyze a human genome for microsatellite content in under 3 min. We also assessed the memory usage of our function and found that memory utilization is minimal. *Clitarchus hookeri* had the largest genome in our benchmark dataset (3.8 Gbp). Peak memory usage for analysis of this genome was 35.5 MB. This level of RAM usage ensures that even large genomic datasets can be analyzed on standard laptops. This is an added benefit of using C to process the sequences since genomes are never stored in memory but rather are read as a stream that minimizes peak memory demands.

**Table 1:** Insect genomes used in benchmarking and testing micRocounter. Assembly size is the size of the assembled genome and not necessarily representative of the true genome size since some assemblies are highly fragmented or missing significant proportions of the genome. All genomes were downloaded from NCBI

Order	Species	Assembly Size (Mbp)	Assembly Version	Accession Number
Blattodea	*Blattella germanica*	2037	1	GCA_003018175.1
Blattodea	*Cryptotermes secundus*	1018	1	GCA_002891405.2
Coleoptera	*Diabrotica virgifera*	2409	2	GCA_003013835.2
Coleoptera	*Priacma serrata*	12	1	GCA_000281835.1
Diptera	*Aedes aegypti*	1,383	5	GCA_002204515.1
Diptera	*Drosophila albomicans*	253	1	GCA_000298335.1
Diptera	*Drosophila melanogaster*	144	6+	GCA_000001215.4
Diptera	*Liriomyza trifolii*	69	1	GCA_001014935.1
Diptera	*Megaselia abdita*	412	1	GCA_001015175.1
Hemiptera	*Rhodnius prolixus*	706	1	GCA_000181055.3
Hymenoptera	*Megastigmus dorsalis*	589	1	GCA_900490025.1
Lepidoptera	*Calephelis virginiensis*	855	1	GCA_002245475.1
Lepidoptera	*Vanessa tameamea*	357	1	GCA_002938995.1
Odonata	*Calopteryx splendens*	1,628	1	GCA_002093875.1
Phasmatidae	*Clitarchus hookeri*	3802	1	GCA_002778355.1

In conclusion, micRocounter provides a fast, accurate, and open source software in the R environment that will facilitate analyses of new genomes and comparative analyses of microsatellite content across genomes of many species. Furthermore, because it is in the R environment, it is simple and straightforward for users to take their results and use them in downstream analyses or visualizations that are already available in other packages. All memory usage and processing time calculations were completed on a MacBook Pro with a 2.8 GHz i9 processor and 16 GB of RAM. Scripts were run in RStudio version 1.1.442 using R version 3.5.2 (R Development Core Team 2013; RStudio Team 2015).
